# Association of the Mediterranean Diet With Onset of Diabetes in the Women’s Health Study

**DOI:** 10.1001/jamanetworkopen.2020.25466

**Published:** 2020-11-19

**Authors:** Shafqat Ahmad, Olga V. Demler, Qi Sun, M. Vinayaga Moorthy, Chunying Li, I-Min Lee, Paul M. Ridker, JoAnn E. Manson, Frank B. Hu, Tove Fall, Daniel I. Chasman, Susan Cheng, Aruna Pradhan, Samia Mora

**Affiliations:** 1 Center for Lipid Metabolomics, Brigham and Women’s Hospital, Harvard Medical School, Boston, Massachusetts; 2 Division of Preventive Medicine, Brigham and Women’s Hospital, Harvard Medical School, Boston, Massachusetts; 3 Cardiovascular Division, Brigham and Women’s Hospital, Harvard Medical School, Boston, Massachusetts; 4 Department of Nutrition, Harvard T.H. Chan School of Public Health, Boston, Massachusetts; 5 Department of Medical Sciences, Molecular Epidemiology, Uppsala University, Uppsala, Sweden; 6 Department of Epidemiology, Harvard T.H. Chan School of Public Health, Boston, Massachusetts; 7 Smidt Heart Institute, Cedars-Sinai Medical Center, Los Angeles, California; 8 Framingham Heart Study, Framingham, Massachusetts

## Abstract

**Importance:**

Higher Mediterranean diet (MED) intake has been associated with reduced risk of type 2 diabetes, but underlying biological mechanisms are unclear.

**Objective:**

To characterize the relative contribution of conventional and novel biomarkers in MED-associated type 2 diabetes risk reduction in a US population.

**Design, Setting, and Participants:**

This cohort study was conducted among 25 317 apparently healthy women. The participants with missing information regarding all traditional and novel metabolic biomarkers or those with baseline diabetes were excluded. Participants were invited for baseline assessment between September 1992 and May 1995. Data were collected from November 1992 to December 2017 and analyzed from December 2018 to December 2019.

**Exposures:**

MED intake score (range, 0 to 9) was computed from self-reported dietary intake, representing adherence to Mediterranean diet intake.

**Main Outcomes and Measures:**

Incident cases of type 2 diabetes, identified through annual questionnaires; reported cases were confirmed by either telephone interview or supplemental questionnaire. Proportion of reduced risk of type 2 diabetes explained by clinical risk factors and a panel of 40 biomarkers that represent different physiological pathways was estimated.

**Results:**

The mean (SD) age of the 25 317 female participants was 52.9 (9.9) years, and they were followed up for a mean (SD) of 19.8 (5.8) years. Higher baseline MED intake (score ≥6 vs ≤3) was associated with as much as a 30% lower type 2 diabetes risk (age-adjusted and energy-adjusted hazard ratio, 0.70; 95% CI, 0.62-0.79; when regression models were additionally adjusted with body mass index [BMI]: hazard ratio, 0.85; 95% CI, 0.76-0.96). Biomarkers of insulin resistance made the largest contribution to lower risk (accounting for 65.5% of the MED–type 2 diabetes association), followed by BMI (55.5%), high-density lipoprotein measures (53.0%), and inflammation (52.5%), with lesser contributions from branched-chain amino acids (34.5%), very low-density lipoprotein measures (32.0%), low-density lipoprotein measures (31.0%), blood pressure (29.0%), and apolipoproteins (23.5%), and minimal contribution (≤2%) from hemoglobin A_1c_. In post hoc subgroup analyses, the inverse association of MED diet with type 2 diabetes was seen only among women who had BMI of at least 25 at baseline but not those who had BMI of less than 25 (eg, women with BMI <25, age- and energy-adjusted HR for MED score ≥6 vs ≤3, 1.01; 95% CI, 0.77-1.33; *P* for trend = .92; women with BMI ≥25: HR, 0.76; 95% CI, 0.67-0.87; *P* for trend < .001).

**Conclusions and Relevance:**

In this cohort study, higher MED intake scores were associated with a 30% relative risk reduction in type 2 diabetes during a 20-year period, which could be explained in large part by biomarkers of insulin resistance, BMI, lipoprotein metabolism, and inflammation.

## Key Points

**Question:**

Is the Mediterranean (MED) diet associated with reduced risk of diabetes in a US population, and if so, what are possible underlying biological mechanistic pathways?

**Findings:**

Among 25 317 women followed up for 20 years in a prospective epidemiological cohort study, 2307 developed type 2 diabetes. Higher baseline MED intake was associated with a 30% reduction in future risk of diabetes; biomarkers of insulin resistance, adiposity, high-density lipoprotein, and inflammation contributed most to explaining this inverse association.

**Meaning:**

These findings suggest that the MED diet may be protective against diabetes by improving insulin resistance, lipoprotein metabolism, and inflammation.

## Teaser

This cohort study characterizes the relative association of conventional and novel biomarkers in Mediterranean diet–related type 2 diabetes risk reduction in a US population.

## Introduction

Overall dietary intake modification, compared with individual dietary attributes, is proposed as a more effective approach for cardiometabolic disease prevention and intervention.^1^ The Mediterranean diet (MED) uses olive oil as the predominant oil; is rich in fruits, vegetables, legumes, nuts, and seeds; includes moderate amounts of fish and dairy products; and is limited in red and processed meats and sweets. Results from observational studies^2,3,4^ and a randomized clinical trial^5^ have provided evidence of a beneficial association of MED intake with reduced risk of type 2 diabetes. The Prevención con Dieta Mediterránea (PREDIMED) randomized trial conducted in Spain reported that the MED intake (pooled supplementation of olive oil and nuts groups) compared with the control group led to 53% reduced risk of incident diabetes during a 4-year period.^6,7^

MED intake has been associated with improvement in multiple cardiometabolic biomarkers, including insulin resistance and hemoglobin A_1c_ (HbA_1c_).^8,9^ In a 4-year follow-up of PREDIMED study, MED intake improved intermediate risk factors in cardiometabolic disease including increasing the number of large high-density lipoprotein (HDL) particles, lowering diastolic blood pressure (but not systolic blood pressure), and significantly reducing low-density lipoprotein (LDL) oxidation and cellular lipid levels. After a 3-month follow-up period in PREDIMED, MED intake improved blood pressure, insulin sensitivity, lipid profile, and circulating inflammatory molecules.^10^ A neutral or marginally beneficial association with weight gain and central adiposity has been observed with interventions in PREDIMED and other studies,^11,12^ and it is unclear whether some of the benefit of MED intake on lower type 2 diabetes risk may be mediated through adiposity measures.

No randomized trials of MED intervention have been conducted in the United States for clinical endpoints such as type 2 diabetes. Observational studies in US populations with a follow-up of 20 years or less and approximately 6 years have reported that adherence to healthy lifestyles, including MED intake, was associated with reduced risk of type 2 diabetes^13^ and improved glycemic biomarkers,^14^ respectively. Furthermore, the precise mechanisms through which MED intake may reduce risk of type 2 diabetes are not well understood, in particular for the relative contribution of traditional and newly discovered biomarkers, particularly those relating to glucose metabolism and insulin resistance, inflammation, novel lipoproteins, and small metabolites. Therefore, the goal of the current study was to examine the association of MED intake and risk of incident diabetes and, importantly, to understand the relative importance of various biological pathways of risk through which MED intake may be associated with lower risk of diabetes. Therefore, in a US population of 25 317 initially healthy women with long-term follow-up, we aimed to examine whether MED intake was associated with lower risk of incident type 2 diabetes and to quantify the contribution of traditional and novel biological factors to the MED-associated reduction in type 2 diabetes risk.

## Methods

### Study Population

Study participants are enrolled in the Women’s Health Study (WHS), a completed clinical trial of vitamin E and low-dose aspirin among initially healthy women free from cardiovascular disease and cancer at baseline.^15,16^ At the time of enrollment, all participants provided informed consent, completed food frequency questionnaires (FFQs) about dietary intake, and answered questions regarding lifestyle factors, demographic characteristics, medical history, and anthropometrics. In the WHS, 28 345 women provided voluntary baseline blood samples. Self-reported body height and weight were obtained from the baseline questionnaires and used to calculate body mass index (BMI; calculated as weight in kilograms divided by height in meters squared), as has been reported previously.^17^ For the current analysis, 25 317 study participants were included because 2371 had missing information on all the traditional and novel metabolic biomarkers (ie, the 40 biomarkers used for the current analysis) and 657 participants had baseline diabetes. This study was approved by the ethical review board at Brigham and Women’s Hospital, Boston. This study followed the Strengthening the Reporting of Observational Studies in Epidemiology (STROBE) reporting guideline.

### MED Intake Assessment

We previously described in detail the method for assessment of the MED score.^18^ Briefly, the MED score (range, 0 to 9) is based on adherence to 9 dietary components; 1 point is given if the intake of that particular dietary component was greater than the study median for intake of fruits, vegetables (excluding potatoes), whole grains, legumes, nuts, fish, and the ratio of mono-unsaturated to saturated fatty acids. For alcohol intake, 1 point was given if the intake was in the range of 5 to 15 g/d, and for dietary red and processed meat intake, 1 point was given if the dietary intake was less than the study median. For the current analysis, MED score was categorized into 3 levels (0-3, 4-5, and 6-9) to allow for comparison with prior studies.^18,19^ Furthermore, the top category (ie, score 6-9) also represents the top quartile of the study population.

### Incident Type 2 Diabetes Ascertainment

All participants were continuously followed up for the occurrence of incident diabetes via annual questionnaires asking whether and when they had been diagnosed with diabetes since baseline. Reported cases of diabetes were confirmed by either telephone interview or supplemental questionnaire. Laboratory data were unavailable to distinguish type 1 diabetes or other diabetes variants. However, because most diabetes diagnosed at age 45 years or older is of the type 2 variant, incident diabetes in the WHS cohort is considered type 2 diabetes.^20^

### Measurement of Traditional Biomarkers

The dietary measures and blood for biomarker measures were taken at the study entry at baseline. At baseline, blood was collected using EDTA tubes, which were stored at −170 °C until analyses were performed. HbA_1c_ was measured with an immunoturbidometric assay (Roche Diagnostics).^21^ Lipoprotein (a) (Lp[a]) and high-sensitivity C-reactive protein (hsCRP) were measured with immunoturbidometric assays using Hitachi-911 analyzer (Roche Diagnostics).^21^ Traditional lipids including total cholesterol, HDL and LDL cholesterol, and triglycerides (TG) were enzymatically assessed (Roche Diagnostics), and TG was corrected for endogenous glycerol. Lipoproteins including apolipoprotein (apo) A1 and B100 were measured using turbidometric assays (Diasorin). Soluble intracellular adhesion molecule 1 (sICAM-1) was quantified using enzyme-linked immunosorbent assay (R&D Systems). Homocysteine was enzymatically assessed using Hitachi-917 analyzer (Roche Diagnostics). Creatinine was assessed through the Jaffe reaction based rate-blanked method (Roche Diagnostics).

### Measurement of Nuclear Magnetic Resonance Biomarkers

Lipoprotein subfraction particles of HDL, LDL, and very low-density lipoprotein (VLDL) biomarkers and small metabolites were measured with targeted nuclear magnetic resonance (NMR) spectroscopy^18,22,23,24^ using 1H-NMR (400 MHz) LipoProfile-IV (LipoScience; now LabCorp). The Lipoprotein Insulin Resistance Index (LPIR) is an NMR-based insulin resistance score that includes subfractions of triglyceride-rich lipoproteins (TRLP), HDL, and LDL particles.^18^ The NMR assay was also used to measure glycoprotein acetylation, which is an aggregate biomarker of circulating glycosylated acute phase proteins and a measure of inflammation, alanine, citrate, and branched-chain amino acids (BCAA [leucine, isoleucine, valine]). The NMR assay was also used to calculate a diabetes risk index (DRI), a multimarker score (range, 1-100) that combines LPIR and BCAA levels.

### Statistical Analysis

Statistical analyses were performed using SAS version 9.3 (SAS Institute) and Stata version 14.0 (StataCorp) software. Cox proportional hazards regression models were used to compute hazard ratios (HRs) with corresponding 95% CIs using the participants with lower MED intake (score 0-3) as the reference category. We used the median value of each of the 3 MED categories (0-3, 4-5, and 6-9) to assess for linear trends (*P* for trend). HRs were estimated among participants who did not yet experience the event of interest or a competing event (eg, mortality).^25^ We considered a 2-sided *P* < .01 as a significant threshold for the biomarker associations with diabetes risk and *P* > .05 for the mediation analysis. To assess the linear trends, median values of each MED intake group were used. Biomarkers of TG, homocysteine, hsCRP, and Lp(a) were not normally distributed, so they were log transformed.

The Baron and Kenny approach was used to test whether all biomarkers satisfied the criteria to be used as mediators, and mediation analysis was performed using the standard mediation approach.^26^ We computed person-years of follow-up time from baseline until diabetes diagnosis or censoring. We first tested significance of association of MED intake with type 2 diabetes. Then, using a separate model for each potential risk biomarker, we again tested for the association of MED intake with type 2 diabetes. All models were adjusted for age, randomized treatment assignment, and energy intake, whereby we evaluated the magnitude of the change in the HRs for the highest vs lowest MED intake group, with and without adjustment for each biomarker. A larger change in the HR toward the null implies a larger mediating effect of the risk factor on the MED intake–associated reduction in type 2 diabetes risk.

Then, on a priori hypothesis, we grouped biomarkers based on their potential physiological functions. Details about these groups have also been discussed previously.^18^ Traditional lipids, including HDL, LDL, total cholesterol, and TG, were grouped together, while apo A1, apo B100, and Lp(a) were grouped together. The biomarkers of sICAM-1, hsCRP, fibrinogen, and glycoprotein acetylation were combined considering their role in inflammation. Biomarkers of apo B100, LDL particles and concentrations, and total LDL were combined as the LDL set. The HDL set contained apo A1, HDL particles and concentration, and HDL cholesterol. We combined TG-lipoprotein particle size and concentration as well as TG in the VLDL set. Small metabolites included citrate, creatinine, homocysteine, and alanine. The total BCAA group was analyzed separately. The hypertension group included systolic and diastolic blood pressure as well as hypertension.

We assessed the magnitude of the change in the HRs comparing highest vs lowest MED intake by adding the potential groups 1 by 1 to the Cox models. The basic model included the basic covariates (ie, age, randomization treatment assignment, energy intake, postmenopausal status, postmenopausal hormone use, smoking, and exercise). To examine the extent to which each set of risk factors potentially mediated the association of MED intake on incident type 2 diabetes, we next added these sets, 1 at a time, to the basic model and examined the magnitude of change in the HRs for the group with the highest MED intake compared with the lowest both without (basic model) and with adjustment for each set (adjusted model = basic model + mediator set). A larger change in the HRs toward null implies a greater mediating effect regarding the association between MED and type 2 diabetes risk. The proportion of diabetes risk reduction explained by each group of mediators was calculated using the following formula: HR_basic model_ − HR_adjusted model_) / (HR_basic model_ − 1) × 100%.^27^ Sensitivity analyses were also performed using counterfactual framework approach.^28,29^ For single biomarker analyses, we used both mediation approaches (standard method and counterfactual framework), and results were similar using both methods. A limitation of the counterfactual framework approach is that it cannot incorporate multiple biomarkers as mediators in a model. Hence, multiple biomarker mediation analyses were only performed using the standard mediation approach.

## Results

### Characteristics

In this study of 25 317 participants free of type 2 diabetes at baseline, and the mean (SD) age was 52.9 (9.9) years. Participants with higher baseline MED intake had a higher intake of fruits, legumes, whole grains, vegetables, nuts, fish, and ratio of monounsaturated to saturated fatty acids and lower consumption of alcohol and red and processed meat intake (*P* for trend < .001) (eTable 1 in the Supplement). Higher MED intake was generally associated with better biomarker profiles, except for 9 biomarkers that were similar across MED categories, including HbA_1c_, which did not differ across categories of MED intake (Table 1).

**Table 1.** Baseline Biomarker Levels According to MED Intake

Biomarker	MED intake, median (IQR)			*P* value for trend
	MED score 0-3 (n = 9873)	MED score 4-5 (n = 9184)	MED score ≥6 (n = 6260)	
Blood pressure, mm Hg				
Systolic	125.0 (115.0-135.0)	125.0 (115.0-135.0)	125.0 (115.0-135.0)	.36
Diastolic	80.0 (70.0-80.0)	80.0 (70.0-80.0)	80.0 (70.0-80.0)	.03
Lipids, cholesterol, mg/dL				
LDL	121.6 (100.7-144.0)	121.1 (100.9-144.1)	121.4 (100.3-144.7)	.88
HDL	51.5 (43.0-61.6)	52.7 (43.9-63.1)	53.8 (44.9-64.5)	<.001
Triglycerides	117.0 (83.0-171.0)	117.0 (83.0-172.0)	116.0 (83.0-167.0)	.09
Total	207.0 (183.0-234.0)	208.0 (184.0-235.0)	208.0 (184.0-236.0)	.003
Lipoproteins, mg/dL				
Lipoprotein(a)	10.4 (4.4-32.3)	10.9 (4.7-33.5)	10.8 (4.5-32.9)	.08
Apolipoprotein A1	148.0 (131.3-166.5)	150.0 (133.7-168.5)	152.0 (135.3-171.0)	<.001
Apolipoprotein B100	99.8 (83.8-120.4)	99.5 (83.2-120.1)	99.2 (83.5-120.5)	.60
LDL particles and size				
LDL particle concentration, nmol/L	1561.0 (1326.0-1831.0)	1562.0 (1327.0-1830.0)	1565.0 (1324.0-1827.0)	.71
LDL particle size, nm	20.9 (20.6-21.2)	20.9 (20.6-21.2)	20.9 (20.6-21.2)	<.001
HDL particles and size				
HDL particle concentration, μmol/L	24.2 (21.8-26.8)	24.4 (22.1-27.1)	24.6 (22.2-27.3)	<.001
HDL particle size, nm	8.9 (8.7-9.2)	8.9 (8.7-9.2)	8.9 (8.7-9.2)	<.001
VLDL measures				
TRL particle concentration, nmol/L	166.7 (131.8-207.3)	165.9 (129.2-208.1)	164.8 (129.1-206.6)	.10
TRL particle size, nm	42.4 (38.5-47.9)	42.5 (38.6-47.7)	42.2 (38.4-47.5)	.08
Glycemic				
Hemoglobin A_1c_, % of total hemoglobin	4.99 (4.8-5.2)	4.99 (4.8-5.2)	4.99 (4.8-5.2)	.005
Insulin resistance				
Lipoprotein insulin resistance index score^a^	40.0 (21.0-62.0)	39.0 (20.0-60.0)	38.0 (20.0-57.0)	<.001
5-y diabetes risk factor index score^a^	41.5 (28.2-54.8)	40.2 (28.2-53.4)	39.4 (27.9-52.0)	<.001
Inflammation				
High-sensitivity C-reactive protein, mg/dL	0.20 (0.08-0.44)	0.20 (0.08-0.42)	0.18 (0.07-0.39)	<.001
Fibrinogen, mg/dL	350.4 (306.7-403.2)	349.7 (307.5-400.1)	346.1 (304.6-396.7)	.001
Soluble intercellular adhesion molecule 1, ng/mL	344.4 (301.5-397.5)	341.2 (299.8-390.6)	337.1 (297.3-383.5)	<.001
Glycoprotein acetylation, μmol/L	384.0 (340.0-432.0)	381.0 (339.0-428.0)	378.0 (335.0-422.0)	<.001
Branched-chain amino acids, μmol/L				
Total branched-chain amino acids, μmol/L	402.0 (350.0-461.0)	398.0 (348.0-456.0)	396.0 (347.0-452.0)	<.001
Valine, mg/dL	2.6 (2.2-2.9)	2.6 (2.3-2.9)	2.5 (2.2-2.9)	.001
Leucine, mg/dL	1.7 (1.4-2.0)	1.7 (1.4-2.0)	1.7 (1.4-2.0)	.007
Isoleucine, mg/dL	0.7 (0.5-0.9)	0.7 (0.5-0.8)	0.6 (0.5-0.8)	<.001
Small molecule metabolites				
Alanine, mg/dL	3.6 (3.0-4.3)	3.6 (3.0-4.2)	3.6 (3.0-4.2)	.14
Citrate, mg/dL	1.8 (1.5-2.1)	1.8 (1.5-2.1)	1.8 (1.5-2.1)	.56
Creatinine, mg/dL	0.7 (0.6-0.8)	0.7 (0.6-0.8)	0.7 (0.6-0.8)	.14
Homocysteine, mg/L	1.4 (1.2-1.8)	1.4 (1.2-1.7)	1.4 (1.2-1.7)	<.001

Abbreviations: HDL, high-density lipoprotein; IQR, interquartile range; LDL, low-density lipoprotein; MED, Mediterranean diet; TRL, triglyceride-rich lipoprotein; VLDL, very low-density lipoprotein.

SI conversion factors: To convert alanine to micromoles per liter, multiply by 112.2; apolipoprotein A1 and B-100 to grams per liter, multiply by 0.01; citrate to micromoles per liter, multiply by 52.05; creatinine to micromoles per liter, multiply by 88.4; fibrinogen to gram per liter, multiply by 0.01; HDL and LDL to millimoles per liter, multiply by 0.0259; hemoglobin A_1c_ to proportion of total hemoglobin, multiply by 0.01; high-sensitivity C-reactive protein to milligrams per liter, multiply by 10; homocysteine to micromoles per liter, multiply by 7.397; lipoprotein(a) to milligrams per liter, multiply by 0.1; isoleucine and leucine to micromoles per liter, multiply by 76.237; triglycerides to millimoles per liter, multiply by 0.0113; valine to micromoles per liter, multiply by 85.361.

^a^ Five-year diabetes risk factor index and lipoprotein insulin resistance index are scored on a scale of 1 to 100, with higher numbers indicating higher risk.

### MED Intake and Risk of Type 2 Diabetes

**Figure 1.**
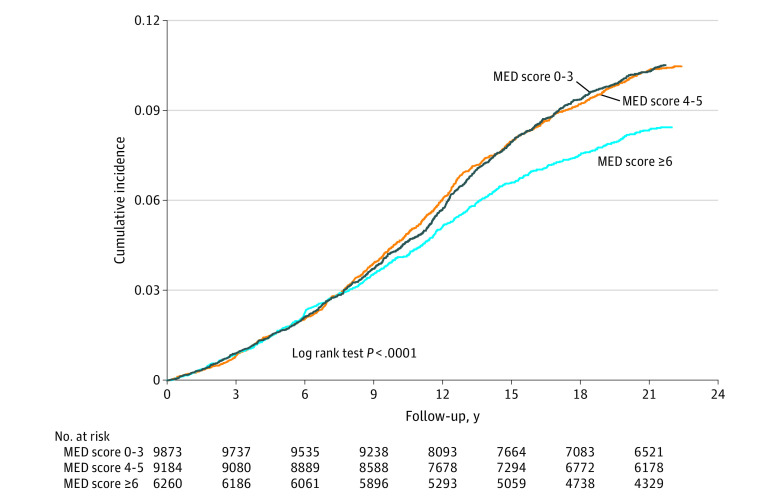
Cumulative Incidence of Type 2 Diabetes by Mediterranean Diet (MED) Intake Groups

A total of 2307 participants developed type 2 diabetes during a mean (SD) follow-up of 19.8 (5.8) years (maximum, 25 years). The diabetes incidence rate was 0.46 (95% CI, 0.44-0.48) per 100 person-years. Higher baseline MED intake (score ≥6 vs ≤3) was significantly associated with 30% lower type 2 diabetes risk (HR, 0.70; 95% CI, 0.62-0.79) (Table 2). The association of the 3 groups of MED intake with type 2 diabetes risk is depicted through cumulative incidence curves in Figure 1. Participants in the highest MED intake group (score ≥6) had the best diabetes-free survival, with the curves for that group separating from the lower 2 groups after approximately 10 years of follow-up.

**Table 2.** Association of MED Intake With Incident Type 2 Diabetes After Adjustment for Individual Risk Factors or Biomarkers

Factor or Biomarker	MED score, HR (95% CI)			*P* value for trend
	0-3	4-5	≥6	
Age, treatment, and energy-adjusted model	1 [Reference]	0.94 (0.85-1.03)	0.70 (0.62-0.79)	<.001
Age, treatment, and energy-adjusted model plus each of the following added 1 at a time				
Smoking	1 [Reference]	0.94 (0.85-1.03)	0.70 (0.62-0.79)	<.001
Alcohol consumption	1 [Reference]	0.99 (0.90-1.09)	0.79 (0.70-0.89)	<.001
Blood pressure				
Hypertension	1 [Reference]	0.97 (0.88-1.06)	0.73 (0.65-0.82)	<.001
Systolic blood pressure	1 [Reference]	0.99 (0.90-1.09)	0.76 (0.67-0.85)	<.001
Diastolic blood pressure	1 [Reference]	0.98 (0.89-1.08)	0.75 (0.66-0.84)	<.001
Traditional lipids, cholesterol				
LDL	1 [Reference]	0.94 (0.86-1.04)	0.70 (0.62-0.79)	<.001
HDL	1 [Reference]	1.03 (0.93-1.13)	0.82 (0.73-0.92)	.003
Triglycerides	1 [Reference]	0.96 (0.87-1.06)	0.75 (0.67-0.85)	<.001
Total	1 [Reference]	0.94 (0.85-1.03)	0.70 (0.62-0.78)	<.001
Lipoproteins				
Lipoprotein(a)	1 [Reference]	0.94 (0.85-1.03)	0.70 (0.62-0.79)	<.001
Apolipoprotein A1	1 [Reference]	0.97 (0.88-1.07)	0.75 (0.66-0.84)	<.001
Apolipoprotein B100	1 [Reference]	0.96 (0.87-1.06)	0.73 (0.65-0.82)	<.001
LDL particles and size				
LDL particle concentration	1 [Reference]	0.95 (0.87-1.05)	0.72 (0.64-0.81)	<.001
LDL particle size	1 [Reference]	0.95 (0.86-1.05)	0.74 (0.65-0.83)	<.001
HDL particles and size				
HDL particle concentration	1 [Reference]	0.95 (0.86-1.04)	0.71 (0.63-0.80)	<.001
HDL particle size	1 [Reference]	1.01 (0.92-1.11)	0.81 (0.71-0.91)	.001
VLDL measures				
TRL particle concentration	1 [Reference]	0.95 (0.86-1.05)	0.72 (0.64-0.81)	<.001
TRL particle size	1 [Reference]	0.95 (0.87-1.05)	0.73 (0.65-0.83)	<.001
Glycemic				
Hemoglobin A_1c_	1 [Reference]	0.91 (0.82-1.00)	0.70 (0.62-0.79)	<.001
Insulin resistance				
Lipoprotein insulin resistance index score	1 [Reference]	1.00 (0.91-1.10)	0.82 (0.73-0.92)	.003
5-y diabetes risk factor index score	1 [Reference]	1.01 (0.92-1.11)	0.82 (0.72-0.92)	.003
Inflammation				
High-sensitivity C-reactive protein	1 [Reference]	0.98 (0.89-1.08)	0.79 (0.70-0.89)	<.001
Fibrinogen	1 [Reference]	0.96 (0.87-1.05)	0.73 (0.65-0.83)	<.001
Soluble intercellular adhesion molecule 1	1 [Reference]	1.00 (0.91-1.10)	0.77 (0.68-0.87)	<.001
Glycoprotein acetylation	1 [Reference]	0.98 (0.89-1.08)	0.78 (0.69-0.88)	<.001
Branched-chain amino acids				
Total branched-chain amino acids	1 [Reference]	0.97 (0.88-1.07)	0.75 (0.66-0.84)	<.001
Valine	1 [Reference]	0.97 (0.89-1.07)	0.75 (0.66-0.84)	<.001
Leucine	1 [Reference]	0.95 (0.86-1.04)	0.72 (0.64-0.81)	<.001
Isoleucine	1 [Reference]	0.98 (0.89-1.08)	0.76 (0.67-0.85)	<.001
Small molecule metabolites				
Citrate	1 [Reference]	0.93 (0.85-1.03)	0.70 (0.62-0.79)	<.001
Creatinine	1 [Reference]	0.94 (0.85-1.03)	0.70 (0.62-0.79)	<.001
Alanine	1 [Reference]	0.94 (0.86-1.04)	0.70 (0.62-0.79)	<.001
Homocysteine	1 [Reference]	0.94 (0.85-1.03)	0.70 (0.62-0.79)	<.001

Abbreviations: HDL, high-density lipoprotein; HR, hazard ratio; LDL, low-density lipoprotein; MED, Mediterranean diet; TRL, triglyceride-rich lipoprotein; VLDL, very low-density lipoprotein.

All studied biomarkers (Table 1) met the criteria for Baron and Kenny for mediation,^26^ except for systolic blood pressure, LDL cholesterol, TGs, LDL particle concentration, TG-rich lipoprotein particle concentration, TG-rich lipoprotein particle size, Lp(a), Apo B100, alanine, citrate, and creatinine. However, a significant inverse association between MED intake and these biomarkers has been reported previously.^30,31,32^ Therefore, in our current analysis, we also included these biomarkers for further analysis (Table 2 and Table 3; eTable 2 in the Supplement). In separate Cox models that were adjusted for age and energy intake, we additionally adjusted for an individual biomarker at time (Table 2). We observed notable attenuation of HRs through comparing higher vs lower MED groups with and without adjustment (in separate models) for BMI as well as for the biomarkers of HDL, insulin resistance, and inflammation, among others.

**Table 3.** Association of MED Intake With Incident Type 2 Diabetes After Adjustment for Sets of Potential Mediators

Factor	MED score, HR (95% CI)			*P* value for trend
	0-3	4-5	≥6	
Age, treatment, and energy-adjusted model	1 [Reference]	0.94 (0.85-1.03)	0.70 (0.62-0.79)	<.001
Basic model^a^	1 [Reference]	1.00 (0.91-1.10)	0.80 (0.71-0.90)	.001
Basic model plus each set of risk factors below, added 1 group at a time^b^				
Insulin resistance: lipoprotein insulin resistance index score, diabetes risk index score	1 [Reference]	1.07 (0.97-1.18)	0.93 (0.82-1.05)	.43
BMI	1 [Reference]	1.08 (0.98-1.19)	0.91 (0.81-1.03)	.80
HDL measure: HDL particle size and concentration, HDL cholesterol, apolipoprotein A1	1 [Reference]	1.08 (0.98-1.19)	0.91 (0.80-1.02)	.25
Inflammation: hsCRP, fibrinogen, sICAM-1, glycoprotein acetylation	1 [Reference]	1.07 (0.97-1.18)	0.91 (0.80-1.02)	.22
Branched-chain amino acids: valine, leucine, isoleucine	1 [Reference]	1.06 (0.96-1.16)	0.87 (0.77-0.98)	.06
VLDL measures: triglyceride-rich lipoprotein particle size and concentrations, triglycerides	1 [Reference]	1.03 (0.93-1.13)	0.86 (0.77-0.98)	.04
LDL measures: LDL particle size and concentration, LDL cholesterol, apolipoprotein B100	1 [Reference]	1.03 (0.94-1.13)	0.86 (0.76-0.97)	.04
Hypertension: history of hypertension, systolic and diastolic blood pressure	1 [Reference]	1.05 (0.95-1.16)	0.86 (0.76-0.97)	.04
Apolipoproteins: lipoprotein(a), apolipoprotein A1, apolipoprotein B100	1 [Reference]	1.04 (0.94-1.14)	0.85 (0.75-0.96)	.02
Hemoglobin A_1c_, %	1 [Reference]	0.98 (0.89-1.08)	0.80 (0.71-0.90)	<.001
Small-molecule metabolites: citrate, creatinine, homocysteine, alanine	1 [Reference]	1.01 (0.92-1.11)	0.82 (0.72-0.92)	.003

Abbreviations: BMI, body mass index; HDL, high-density lipoprotein; HR, hazard ratio; hsCRP, high sensitivity C-reactive protein; LDL, low-density lipoprotein; MED, Mediterranean diet; sICAM-1, soluble intercellular adhesion molecule 1; VLDL, very low-density lipoproteins.

^a^ Basic model included age, randomized treatment assignment, energy intake, smoking, menopausal status, postmenopausal hormone use, and physical activity.

^b^ Models were adjusted for the variables in the basic model plus each of the sets of risk factors added 1 group at a time to separate models.

### Adjustment for Each Set of Intermediate Biomarkers on the MED–Type 2 Diabetes Association

Next, to determine the extent to which the reduced risk of type 2 diabetes associated with MED intake was explained by potential mediators representing various physiological pathways, each set of mediators was added, 1 set at a time, to the basic model (Table 3). These sets of biomarkers were grouped based on their potential physiological functions. The addition of biomarkers of insulin resistance to the basic model attenuated the inverse relation, which became nonsignificant (MED score, 4-5: HR, 1.07; 95% CI, 0.97-1.18; MED score, ≥6: HR, 0.93; 95% CI, 0.82-1.05; *P* for trend = .43). Similar findings were observed in separate models for BMI (MED score, 4-5: HR, 1.08; 95% CI, 0.98-1.19; MED score, ≥6: HR, 0.91; 95% CI, 0.81-1.03; *P* for trend = .80), HDL measures (MED score, 4-5: HR, 1.08; 95% CI, 0.98-1.19; MED score, ≥6: HR, 0.91; 95% CI, 0.80-1.02; *P* for trend = .25), and inflammation (MED score, 4-5: HR, 1.07; 95% CI, 0.97-1.18; MED score, ≥6: HR, 0.91; 95% CI, 0.80-1.02; *P* for trend = .22). BMI showed significant association with other biomarkers (eTable 6 in the Supplement). The addition of the following sets of intermediate biomarkers (also 1 set at a time) resulted in smaller attenuation: BCAA, VLDL measures, LDL measures, hypertension, and apolipoproteins. No attenuation was observed for the other small molecule metabolites or HbA_1c_ (Table 3).

### Proportion of Reduction of Type 2 Diabetes Risk Explained by Potential Mediators

**Figure 2.**
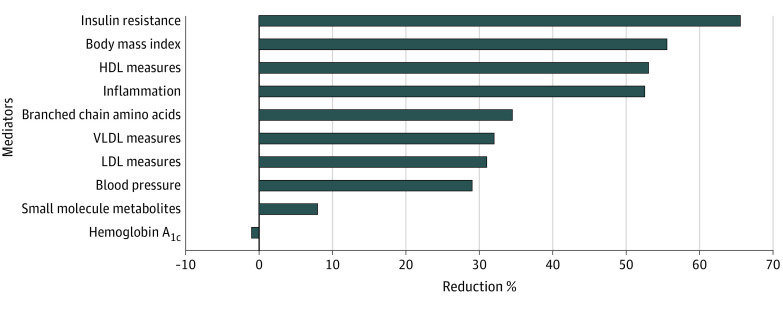
Proportion of Diabetes Risk Reduction for Mediterranean Diet Score of 6 or Greater Proportion was calculated as: HR_basic_ model – HR_adjusted model_) / (HR_basic model_ – 1) × 100%. The basic model included age, randomized treat assignment, energy intake, smoking, menopausal status, postmenopausal hormone use, and physical activity. Insulin resistance included lipoprotein insulin resistance; high density lipoprotein (HDL) measures included HDL cholesterol, HDL particle size and particle concentration, and apolipoprotein A1; inflammation included fibrinogen, high-sensitivity C-reactive protein, intracellular adhesion molecule 1, and glycoprotein acetylation; branched chain amino acids included valine, leucine, and isoleucine; very low density lipoprotein (VLDL) measures included triglycerides and triglyceride-rich lipoprotein subfraction particle concentration and particle size; LDL measures included LDL cholesterol, LDL particle size and particle concentration, apolipoprotein B100; blood pressure included systolic and diastolic blood pressure as well as hypertension; small molecule metabolites include citrate, alanine, creatinine, and homocysteine.

We then calculated the proportion of MED intake reduction in the type 2 diabetes risk explained by these different sets of biomarkers (Figure 2). Biomarkers of insulin resistance made the largest contribution to MED–type 2 diabetes risk (accounting for 65.5% of the inverse MED–type 2 diabetes association); followed by BMI (55.5%), HDL measures (53.0%), in particular HDL size; and inflammation (52.5%), with lesser contributions observed from BCAA (34.5%), VLDL measures (32.0%), and LDL measures (31.0%) (but not LDL cholesterol), blood pressure (29.0%), and apolipoproteins (23.5%), and minimal contribution from HbA_1c_ (≤2%).

### Sensitivity Analyses

We performed a sensitivity analysis for the single mediator biomarker analysis using the counterfactual framework approach in comparison with the standard mediation approach.^33^ We observed generally similar mediation estimates using both the standard mediation approach as well counterfactual framework approach (eTable 2 and eFigure in the Supplement).

We also adjusted single mediator and group mediator analysis while additionally adjusting for BMI (because BMI substantially attenuated the association of MED diet with type 2 diabetes) and observed materially similar results (eTable 3 and eTable 4 in the Supplement, respectively). We also conducted post hoc stratified analyses by baseline BMI, categorizing participants into 2 groups (BMI, <25 and ≥25). Notably, the inverse association of MED diet with type 2 diabetes was seen only among women who had BMI of at least 25 at baseline but not in women with BMI of less than 25 (eg, women with BMI <25: age- and energy-adjusted HR for MED score ≥6 vs ≤3, 1.01; 95% CI, 0.77-1.33; *P* for trend = .92; women with BMI ≥25: HR, 0.76; 95% CI, 0.67-0.87; *P* for trend < .001) (eTable 5 in the Supplement). Among women with BMI of at least 25, the biomarker mediation associations for MED and type 2 diabetes were similar to the overall results (results not shown).

## Discussion

Previous studies have shown that higher consumption of MED intake is associated with reduced risk of type 2 diabetes, but the underlying biological mechanisms regarding this association are unclear. The current prospective study conducted in more than 25 000 women followed up for as long as 25 years indicates that higher consumption of MED was associated with as much as a 30% lower risk of type 2 diabetes, which can largely be explained by both traditionally measured and novel biomarkers. Biomarkers of insulin resistance made the largest contribution, followed by BMI, HDL measures, and inflammation, with lesser contributions from BCAA, VLDL measures, LDL measures (but not LDL cholesterol), blood pressure, and apolipoproteins. Of note, measures of glycaemia, specifically HbA_1c_, did not contribute to the lower risk of MED with type 2 diabetes.

Our results are consistent with the prior evidence, which suggests that higher consumption of MED intake was associated with 30% lower risk of diabetes,^6,34,35^ while in another US population higher MED intake was associated with 25% reduction in diabetes.^13^ Previous studies have demonstrated a favorable effect of MED intake on metabolic biomarkers.^3,4,36,37^ The effect of MED intake on insulin resistance–related biomarkers, including fasting insulin and fasting glucose, have been reported.^3^ We have previously reported that MED intake is associated with improved metabolic, inflammatory, insulin resistance, and adiposity biomarkers^18^ and lower cardiovascular disease risk, by up to 28%. Our current findings support that insulin resistance, adiposity, lipoprotein metabolism, and inflammation are the most relevant contributors to the inverse MED–diabetes risk association. MED is traditionally a plant-based diet with relatively high consumption of extra virgin olive oil and less consumption of red meat and sweets. Hence, this complex nutrient density with low glycemic index may explain the lower risk of type 2 diabetes.

### Strengths and Limitations

Strengths of the study include the prospective epidemiological design with detailed dietary intake measures, a large number of incident diabetes cases during 25 years of follow-up, and comprehensive traditional and NMR-based novel biomarkers assessments. There are some limitations that need to be acknowledged. Study participants were well-educated female health professionals across the United States who were predominantly White individuals and might have different behaviors than men, individuals from other racial/ethnic backgrounds, or the general public. Studies that stratified by ethnicity have reported that MED diet is associated with improved glycemic control and reduced cardiovascular disease risk, blood pressure, and obesity among Black individuals.^38^ The possibility of residual confounding regarding unmeasured factors cannot be ruled out. In the present study, dietary information was assessed through self-reported FFQs, which might lead to the possibility of exposure misclassification, underreporting and overreporting that might attenuate the MED-diabetes association toward null. Other key limitations include the fact that BMI was self-reported, participants were not uniformly screened for diabetes and surveillance bias might be possible, and the current study was not a randomized clinical trial. Inherent to observational epidemiological study design is the possibility of residual confounding of unmeasured factors. However, the models included detailed adjustment with potential confounders, and additional adjustment for other factors made only negligible changes in the estimates, which suggests that residual confounding is unlikely. Furthermore, the results from the counterfactual framework approach were similar to the standard approach, and the MED diet score has the potential to minimize confounding by including nutritional confounders in the score and capturing effect modification among the nutritional variables.^39^ Diet intake was only examined at study entry to be consistent with the time of biomarker measurements. Diet was self-reported and was assessed through a validated FFQ, which reduces the possibility of measurement error. As single measurements were performed for both dietary intake and biomarkers, it is possible that some of the covariates examined (ie, hypertension or BMI) may have influenced subsequent consumption of MED intake, suggesting that they could be both intermediate factors and/or confounders. Finally, multiple comparisons were performed, increasing the chance of a type I error.

## Conclusions

The findings of this cohort suggest that a proportion of the lower risk of diabetes associated with a Mediterranean-type dietary pattern may be mediated through insulin resistance, BMI, lipoprotein metabolism, and inflammation. In exploratory analyses, the inverse association between MED intake and type 2 diabetes risk was only observed among women with BMI of at least 25. Whether a Mediterranean-type dietary intervention in a US population can reduce risk of cardiometabolic disease remains to be tested in future clinical trials.

## Notes

Supplement. **eTable 1. **Baseline Characteristics According to Mediterranean Diet Intake **eTable 2. **Percentage Reduction in Incident Type 2 Diabetes Associated With Mediterranean Diet That Is Explained by Potential Risk Mediators **eTable 3. **Association of Mediterranean Diet Intake With Incident Type 2 Diabetes After Additional Adjustment for BMI **eTable 4. **Association of Mediterranean Diet With Incident Type 2 Diabetes After Adjustment for Sets of Potential Mediators and BMI **eTable 5. **Association of Mediterranean Diet With Incident Type 2 Diabetes Across Obesity Groups After Adjustment for Sets of Potential Mediators **eTable 6. **Pearson Correlation Between BMI and Biomarkers **eFigure. **Percentage Reduction in Incident Type 2 Diabetes Associated With Mediterranean Diet Explained by Potential Risk Mediators Using the Standard Mediation Approach and Counterfactual Framework Approach
