# Supplementary material for: Association of the Mediterranean Diet With Onset of Diabetes in the Women’s Health Study
Source: JAMA Netw Open. 2020 Nov 19;3(11):e2025466. doi: 10.1001/jamanetworkopen.2020.25466 (PMC7677766; doi:10.1001/jamanetworkopen.2020.25466)
Supplement: Supplement. — eTable 1. Baseline Characteristics According to Mediterranean Diet Intake eTable 2. Percentage Reduction in Incident Type 2 Diabetes Associated With Mediterranean Diet That Is Explained by Potential Risk Mediators eTable 3. Association of Mediterranean Diet Intake With Incident Type 2 Diabetes After Additional Adjustment for BMI eTable 4. Association of Mediterranean Diet With Incident Type 2 Diabetes After Adjustment for Sets of Potential Mediators and BMI eTable 5. Association of Mediterranean Diet With Incident Type 2 Diabetes Across Obesity Groups After Adjustment for Sets of Potential Mediators eTable 6. Pearson Correlation Between BMI and Biomarkers eFigure. Percentage Reduction in Incident Type 2 Diabetes Associated With Mediterranean Diet Explained by Potential Risk Mediators Using the Standard Mediation Approach and Counterfactual Framework Approach [file jamanetwopen-e2025466-s001.pdf]

Supplemental Online Content

Ahmad S, Demler OV, Sun Q, et al. Association of the Mediterranean diet with onset of diabetes in the Women's Health Study. *JAMA Netw Open*. 2020;3(11):e2025466. doi:10.1001/jamanetworkopen.2020.25466

eTable 1. Baseline Characteristics According to Mediterranean Diet Intake

eTable 2. Percentage Reduction in Incident Type 2 Diabetes Associated With Mediterranean Diet That Is Explained by Potential Risk Mediators

eTable 3. Association of Mediterranean Diet Intake With Incident Type 2 Diabetes After Additional Adjustment for BMI

eTable 4. Association of Mediterranean Diet With Incident Type 2 Diabetes After Adjustment for Sets of Potential Mediators and BMI

eTable 5. Association of Mediterranean Diet With Incident Type 2 Diabetes Across Obesity Groups After Adjustment for Sets of Potential Mediators

eTable 6. Pearson Correlation Between BMI and Biomarkers

eFigure. Percentage Reduction in Incident Type 2 Diabetes Associated With Mediterranean Diet Explained by Potential Risk Mediators Using the Standard Mediation Approach and Counterfactual Framework Approach

This supplemental material has been provided by the authors to give readers additional information about their work.

© 2020 Ahmad S et al. *JAMA Network Open.*

eTable 1. Baseline Characteristics According to Mediterranean Diet Intake

	Mediterranean Diet Intake		
Characteristic	MED Score 0-3 (n=9,873)a	MED Score 4-5 (n=9,184)	MED Score ≥6 (n=6,263)
Age, median (IQR), y	51.9 (48.4-57.3)	53.3 (49.1-59.1)	54.1 (49.8-60.6)
Current smoking, %	15.7	10.4	6.3
Exercise, %			
Rarely/Never	44.7	35.4	26.0
<1 /week	20.3	19.0	19.2
1-3/week	27.0	33.7	38.5
4+ /week	8.0	11.9	16.3
Alcohol consumption, %			
Rarely	49.2	41.8	34.9
1-3 drinks/month	14.6	13.4	11.0
1–6 drinks/week	27.8	33.8	40.3
≥1 drinks/day	8.4	11.0	13.8
Vegetable intake, median (IQR), servings/day	2.3 (1.6-3.1)	3.7 (2.8-5.0)	5.2 (4.1-6.8)
Fruits, median (IQR), servings/day	1.3 (0.8-1.8)	2.1 (1.4-2.9)	2.8 (2.2-3.7)
Nuts, median (IQR), servings/day	0 (0-0.07)	0.07 (0-0.1)	0.07 (0-0.1)
Whole grains, median (IQR), servings/day	0.7 (0.3-1.1)	1.2 (0.7-1.9)	1.8 (1.3-2.8)
Legumes, median (IQR), servings/day	0.2 (0.1-0.4)	0.4 (0.2-0.6)	0.6 (0.4-0.9)
Fish, median (IQR), servings/day	0.1 (0.07-0.2)	0.2 (0.1-0.3)	0.3 (0.2-0.5)
Ratio of monounsaturated to saturated fat	1.1 (1.0-1.1)	1.1 (1.0-1.2)	1.2 (1.1-1.3)
Red meat, median (IQR), servings/day	0.6 (0.4-1.0)	0.6 (0.3-1.0)	0.5 (0.3-0.9)
Processed meats, median (IQR), servings/day	0.1 (0.1-0.3)	0.1 (0-0.2)	0.07 (0-0.2)
Postmenopausal status, %	49.8	55.3	59.0

Abbreviations: IQR, interquartile range; MED, Mediterranean diet. [a]The MED score is based on 9 components of MED intake. A higher score represents better adherence to the MED, ranging from 0 to 9. For the current analysis, we categorized the participants according to 3 levels of MED (group 1 [lowest]: MED score 0-3; group 2: MED score 4-5; and group 3 [highest]: MED score 6-9).

© 2020 Ahmad S et al. *JAMA Network Open.*

eTable 2. Percentage Reduction in Incident Type 2 Diabetes Associated With Mediterranean Diet That Is Explained by Potential Risk Mediators

	**% Mediation effect explained through standard approach**	**% Mediation effect explained through Counterfactual Framework**
BMI (kg/m^2)	68.1	82.7
Lipoprotein insulin resistance index score	68.1	61.5
5-y diabetes risk factor index score	68.1	63.2
HDL cholesterol, mg/dL	53.2	46.2
High-sensitivity CRP, mg/L	48.9	47.8
Hemoglobin A1c, % of total hemoglobin	-10.6	46.6
HDL particle size, nm	44.7	40.9
Isoleucine, µmol/L	34.0	42.1
Total branched-chain amino acids, (µmol/L)	27.7	36.4
Valine, µmol/L	34.0	35.4
TRL particle size, nm	34.0	34.6
Glycoprotein acetylation , µmol/L	31.9	31.9
Triglycerides, mg/dL	31.9	27.2
Systolic blood pressure, mmHg	29.8	29.3
Diastolic blood pressure, mmHg	25.5	25.3
Soluble ICAM-1, ng/mL	23.4	22.4
LDL particle size, nm	21.3	19
Apolipoprotein A1, mg/dL	19.1	19
Leucine, µmol/L	10.6	14.4
Fibrinogen, mg/dL	8.5	12.6
Alanine, umol/L	6.4	9.7
TRL particle concentration, nmol/L	8.5	9
Apolipoprotein B-100, mg/dL	8.5	5.5
HDL particle concentration, µmol/L	4.3	5.3
LDL particle concentration, nmol/L	4.3	3.6
Citrate (µmol/L)	0.0	2.3
Lipoprotein(a), mg/dL	0.0	1.1
Creatinine, mg/dL	0.0	-0.4
LDL cholesterol, mg/dL	-2.1	-0.7
Homocysteine, umol/L	-2.1	-1.2
Total cholesterol, mg/dL	-2.1	-2.5

Regression model were adjusted for age, randomized treat assignment; total energy intake (TEI, quintiles), smoking, menopausal status, postmenopausal hormone use, physical activity.

© 2020 Ahmad S et al. *JAMA Network Open.*

eTable 3. Association of Mediterranean Diet Intake With Incident Type 2 Diabetes After Additional Adjustment for BMI

	Mediterranean Diet Score			
	HR (95% CI)a			
Biomarker	MED Score 0-3	MED Score 4-5	MED Score ≥ 6	P value for Trend
Age, treatment, and energy-adjusted model	1 [Reference]	1.05 (0.95-1.16)	0.85 (0.76-0.96)	0.03
Age, treatment, and energy-adjusted model plus each of the following added 1 at a time:				
Smoking	1 [Reference]	1.06 (0.96-1.16)	0.87 (0.77-0.98)	0.06
Alcohol consumption	1 [Reference]	1.08 (0.98-1.19)	0.92 (0.81-1.04)	0.32
**Blood pressure**				
Hypertension	1 [Reference]	1.06 (0.97-1.17)	0.87 (0.77-0.98)	0.07
Systolic, mmHg	1 [Reference]	1.07 (0.97-1.17)	0.88 (0.78-0.99)	0.08
Diastolic, mmHg	1 [Reference]	1.07 (0.97-1.17)	0.88 (0.78-1.00)	0.11
**Traditional lipids, cholesterol, mg/dL**				
LDL	1 [Reference]	1.05 (0.95-1.16)	0.86 (0.76-0.97)	0.03
HDL	1 [Reference]	1.09 (0.99-1.20)	0.92 (0.81-1.04)	0.34
Triglycerides	1 [Reference]	1.04 (0.94-1.14)	0.88 (0.78-0.99)	0.08
Total	1 [Reference]	1.05 (0.95-1.15)	0.85 (0.76-0.96)	0.03
**Lipoproteins, mg/dL**				
Lipoprotein(a)	1 [Reference]	1.06 (0.96-1.16)	0.86 (0.76-0.97)	0.04
Apolipoprotein A1	1 [Reference]	1.07 (0.97-1.17)	0.88 (0.78-0.99)	0.09
Apolipoprotein B-100	1 [Reference]	1.05 (0.95-1.16)	0.87 (0.77-0.98)	0.06
**LDL particles and size**				
LDL particle concentration, nmol/L	1 [Reference]	1.05 (0.95-1.15)	0.86 (0.76-0.97)	0.04
LDL particle size, nm	1 [Reference]	1.04 (0.95-1.15)	0.87 (0.77-0.98)	0.05
**HDL particles and size**				
HDL particle concentration, µmol/L	1 [Reference]	1.06 (0.96-1.16)	0.86 (0.76-0.97)	0.04
HDL particle size, nm	1 [Reference]	1.08 (0.98-1.19)	0.92 (0.81-1.04)	0.33
**VLDL measures**	1 [Reference]			
TRL particle concentration, nmol/L	1 [Reference]	1.04 (0.95-1.15)	0.87 (0.77-0.98)	0.05
TRL particle size, nm	1 [Reference]	1.06 (0.96-1.17)	0.89 (0.79-1.00)	0.11
**Glycemic**				
Hemoglobin A1c, % of total hemoglobin	1 [Reference]	1.05 (0.96-1.16)	0.90 (0.79-1.01)	0.15
**Insulin resistance**				
Lipoprotein insulin resistance index score	1 [Reference]	1.07 (0.97-1.17)	0.93 (0.82-1.05)	0.41
5-y diabetes risk factor index score	1 [Reference]	1.09 (0.99-1.20)	0.93 (0.83-1.05)	0.46
**Inflammation**				
High-sensitivity C-reactive protein, mg/L	1 [Reference]	1.06 (0.96-1.16)	0.88 (0.78-0.99)	0.09
Fibrinogen, mg/dL	1 [Reference]	1.05 (0.95-1.16)	0.86 (0.76-0.97)	0.03
Soluble intercellular adhesion molecule 1, ng/mL	1 [Reference]	1.08 (0.98-1.19)	0.90 (0.80-1.02)	0.2
Glycoprotein acetylation , µmol/L	1 [Reference]	1.05 (0.95-1.16)	0.88 (0.79-0.99)	0.08
**Branched-chain amino acids, µmol/L**				

© 2020 Ahmad S et al. *JAMA Network Open.*

Total branched-chain amino acids	1 [Reference]	1.08 (0.98-1.19)	0.90 (0.79-1.01)	0.17
Valine	1 [Reference]	1.08 (0.98-1.19)	0.89 (0.79-1.01)	0.15
Leucine	1 [Reference]	1.07 (0.97-1.17)	0.87 (0.77-0.98)	0.07
Isoleucine	1 [Reference]	1.08 (0.98-1.19)	0.90 (0.80-1.02)	0.22
**Small molecule metabolites**				
Citrate (µmol/L)	1 [Reference]	1.05 (0.95-1.16)	0.86 (0.76-0.97)	0.03
Creatinine, mg/dL	1 [Reference]	1.05 (0.96-1.16)	0.85 (0.76-0.96)	0.03
Homocysteine, umol/L		1.05 (0.95-1.15)	0.85 (0.75-0.96)	0.02
Alanine, umol/L	1 [Reference]	1.05 (0.95-1.16)	0.86 (0.76-0.97)	0.04

Abbreviations: HDL, high-density lipoprotein; HR, hazard ratio; LDL, low-density lipoprotein; MED, Mediterranean diet; TRL, triglyceride-rich lipoprotein; VLDL, very low-density lipoprotein. SI conversion factors: To convert HDL and LDL cholesterol to mmol/L, multiply by 0.0253; triglycerides to mmol/L, multiply by 0.0113; lipoprotein(a) to µmol/L, multiply by 0.0357; apolipoprotein AI and Apolipoprotein B100 to g/L, multiply by 0.01; hemoglobin A1c to proportion of total hemoglobin, multiply by 0.01; C-reactive protein to nmol/L, multiply by 9.524; fibrinogen to µmol/L, multiply by 0.0294; and creatinine to µmol/L, multiply by 88.4. aWe categorized the participants according to 3 levels of MED (scores of 0-3, 4-5, and 6-9). P values across 3 levels of MED were all less than .05.

© 2020 Ahmad S et al. *JAMA Network Open.*

eTable 4. Association of Mediterranean Diet With Incident Type 2 Diabetes After Adjustment for Sets of Potential Mediators and BMI

	Mediterranean Diet			
	MED 0-3	MED 4-5	MED ≥ 6	P for Trend
Age, treatment, and TEI-adjusted model	1 [Reference]	0.94 (0.85-1.03)	0.70 (0.62-0.79)	<0.001
Basic model*	1 [Reference]	1.08 (0.98-1.19)	0.91 (0.81-1.03)	0.28
Basic model plus each set of risk factors below, added 1 group at a time†				
Hypertension: History of hypertension, systolic and diastolic blood pressure	1 [Reference]	1.10 (1.00-1.21)	0.94 (0.83-1.06)	0.57
Hemoglobin A1c, % of total hemoglobin	1 [Reference]	1.09 (0.99-1.20)	0.95 (0.84-1.07)	0.65
Apolipoproteins: lipoprotein(a), apolipoprotein AI, apolipoprotein B100	1 [Reference]	1.09 (0.99-1.20)	0.94 (0.83-1.06)	0.50
LDL measures: LDL particle size and concentration, LDL cholesterol, apolipoprotein B100	1 [Reference]	1.07 (0.98-1.18)	0.94 (0.83-1.07)	0.55
HDL measure: HDL particle size and concentration, HDL cholesterol, apolipoprotein AI	1 [Reference]	1.11 (1.01-1.23)	0.97 (0.86-1.10)	0.98
VLDL measures: triglyceride-rich lipoprotein particle size and concentrations, triglycerides	1 [Reference]	1.08 (0.98-1.19)	0.94 (0.83-1.07)	0.56
Inflammation: hsCRP, fibrinogen, sICAM-1, glycoprotein acetylation	1 [Reference]	1.11 (1.00-1.22)	0.96 (0.85-1.09)	0.86
Insulin resistance: Lipoprotein insulin resistance index score, 5-y diabetes risk factor index score	1 [Reference]	1.12 (1.01-1.23)	1.00 (0.89-1.13)	0.68
Branched-chain amino acids	1 [Reference]	1.12 (1.02-1.23)	0.97 (0.86-1.10)	0.96
Small-molecule metabolites: citrate, creatinine, homocysteine, alanine	1 [Reference]	1.08 (0.98-1.19)	0.92 (0.81-1.04)	0.34

Abbreviations: HDL, high-density lipoprotein; HR, hazard ratio; hsCRP, high sensitivity C-reactive protein; LDL, low-density lipoprotein; sICAM-1, soluble intercellular adhesion molecule 1; VLDL, very low-density lipoproteins. We categorized the participants according to 3 levels of MED (scores of 0-3, 4-5 and 6). P values across 3 levels of MED were all less than .05. *Basic model included age, randomized treatment assignment, energy intake, smoking, menopausal status, postmenopausal hormone use, physical activity. Models were adjusted for the variables in the basic model plus each of the sets of risk factors added 1 group at a time to separate models.

© 2020 Ahmad S et al. *JAMA Network Open.*

eTable 5. Association of Mediterranean Diet With Incident Type 2 Diabetes Across Obesity Groups After Adjustment for Sets of Potential Mediators

	Mediterranean Diet						
		Normal Weight (<25 kg/m²)			Overweight and obese (≥25 kg/m²)		
	MED 0-3	MED 4-5	MED ≥ 6	P for Trend	MED 4-5	MED ≥ 6	P for Trend
Age, treatment, and TEI-adjusted model	1 [Reference]	1.07 (0.85-1.36)	1.01 (0.77-1.33)	0.92	0.97 (0.88-1.08)	0.76 (0.67-0.87)	0.0002
Basic model*	1 [Reference]	1.13 (0.89-1.44)	1.12 (0.85-1.49)	0.40	1.02 (0.92-1.13)	0.84 (0.73-0.96)	0.02
Basic model plus each set of risk factors below, added 1 group at a time†							
Hypertension: History of hypertension, systolic and diastolic blood pressure	1 [Reference]	1.15 (0.91-1.47)	1.14 (0.86-1.51)	0.33	1.05 (0.95-1.17)	0.87 (0.76-1.00)	0.18
Hemoglobin A1c, % of total hemoglobin	1 [Reference]	1.06 (0.83-1.36)	1.13 (0.85-1.50)	0.39	1.02 (0.92-1.14)	0.83 (0.72-0.95)	0.02
Body mass index	1 [Reference]	1.13 (0.89-1.43)	1.14 (0.86-1.51)	0.34	1.07 (0.96-1.18)	0.89 (0.77-1.02)	0.19
Apolipoproteins: lipoprotein(a), apolipoprotein AI, apolipoprotein B100	1 [Reference]	1.17 (0.92-1.48)	1.14 (0.86-1.51)	0.33	1.04 (0.94-1.16)	0.86 (0.75-0.99)	0.08
LDL measures: LDL particle size and concentration, LDL cholesterol, apolipoprotein B100	1 [Reference]	1.14 (0.90-1.45)	1.14 (0.86-1.51)	0.35	1.05 (0.94-1.16)	0.89 (0.78-1.02)	0.18
HDL measure: HDL particle size and concentration, HDL cholesterol, apolipoprotein AI	1 [Reference]	1.20 (0.94-1.52)	1.18 (0.89-1.57)	0.22	1.07 (0.96-1.19)	0.90 (0.78-1.03)	0.25
VLDL measures: triglyceride-rich lipoprotein particle size and concentrations, triglycerides	1 [Reference]	1.15 (0.91-1.47)	1.17 (0.88-1.55)	0.26	1.04 (0.94-1.16)	0.88 (0.77-1.01)	0.12
Inflammation: hsCRP, fibrinogen, sICAM-1, glycoprotein acetylation	1 [Reference]	1.15 (0.91-1.47)	1.16 (0.88-1.54)	0.28	1.07 (0.97-1.19)	0.92 (0.80-1.05)	0.38
Insulin resistance: Lipoprotein insulin resistance index score, 5-y diabetes risk factor index score	1 [Reference]	1.15 (0.91-1.46)	1.20 (0.90-1.59)	0.20	1.08 (0.97-1.20)	0.93 (0.81-1.06)	0.48
Branched-chain amino acids	1 [Reference]	1.15 (0.90-1.46)	1.16 (0.87-1.54)	0.28	1.07 (0.96-1.19)	0.89 (0.78-1.02)	0.21
Small-molecule metabolites: citrate, creatinine, homocysteine, alanine	1 [Reference]	1.14 (0.90-1.45)	1.13 (0.85-1.50)	0.37	1.02 (0.92-1.14)	0.85 (0.74-0.97)	0.04

Abbreviations: HDL, high-density lipoprotein; HR, hazard ratio; hsCRP, high sensitivity C-reactive protein; LDL, low-density lipoprotein; sICAM-1, soluble intercellular adhesion molecule 1; VLDL, very low-density lipoproteins. We categorized the participants according to 3 levels of MED (scores of 0-3, 4-5 and 6). P values across 3 levels of MED were all less than .05. *Basic model included age, randomized treatment assignment, energy intake, smoking, menopausal status, postmenopausal hormone use, physical activity. Models were adjusted for the variables in the basic model plus each of the sets of risk factors added 1 group at a time to separate models.

© 2020 Ahmad S et al. *JAMA Network Open.*

**eTable 6.** Pearson Correlation Between BMI and Biomarkers		
**Biomarkers**	**Correlation coefficient**	**P-value**
**Blood pressure**		
Systolic, mmHg	0.34	<0.001
Diastolic, mmHg	0.30	<0.001
**Traditional lipids, cholesterol, mg/dL**		
LDL	0.12	<0.001
HDL	-0.34	<0.001
Triglycerides	0.26	<0.0001
Total	0.06	<0.001
**Lipoproteins, mg/dL**		
Lipoprotein(a)	0.01	0.33
Apolipoprotein A1	-0.24	<0.001
Apolipoprotein B-100	0.21	<0.001
**LDL particles and size**		
LDL particle concentration, nmol/L	0.19	<0.001
LDL particle size, nm	-0.20	<0.001
**HDL particles and size**		
HDL particle concentration, µmol/L	-0.13	<0.001
HDL particle size, nm	-0.37	<0.001
**VLDL measures**		
TRL particle concentration, nmol/L	0.18	<0.001
TRL particle size, nm	0.25	<0.001
**Glycemic**		
Hemoglobin A1c, % of total hemoglobin	0.22	<0.001
**Insulin resistance**		
Lipoprotein insulin resistance index score	0.40	<0.001
5-y diabetes risk factor index score	0.41	<0.001
**Inflammation**		
High-sensitivity C-reactive protein, mg/L	0.31	<0.001
Fibrinogen, mg/dL	0.33	<0.001
Soluble intercellular adhesion molecule 1, ng/mL	0.18	<0.001
Glycoprotein acetylation , µmol/L	0.38	<0.001
**Branched-chain amino acids, µmol/L**		
Total branched-chain amino acids	0.29	<0.001
Valine	0.31	<0.001
Leucine	0.20	<0.001
Isoleucine	0.24	<0.001
**Small molecule metabolites**		
Citrate, µmol/L	0.03	<0.001
Creatinine, mg/dL	-0.02	0.006
Homocysteine, umol/L	0.03	<0.001
Alanine, umol/L	0.14	<0.001

© 2020 Ahmad S et al. *JAMA Network Open.*

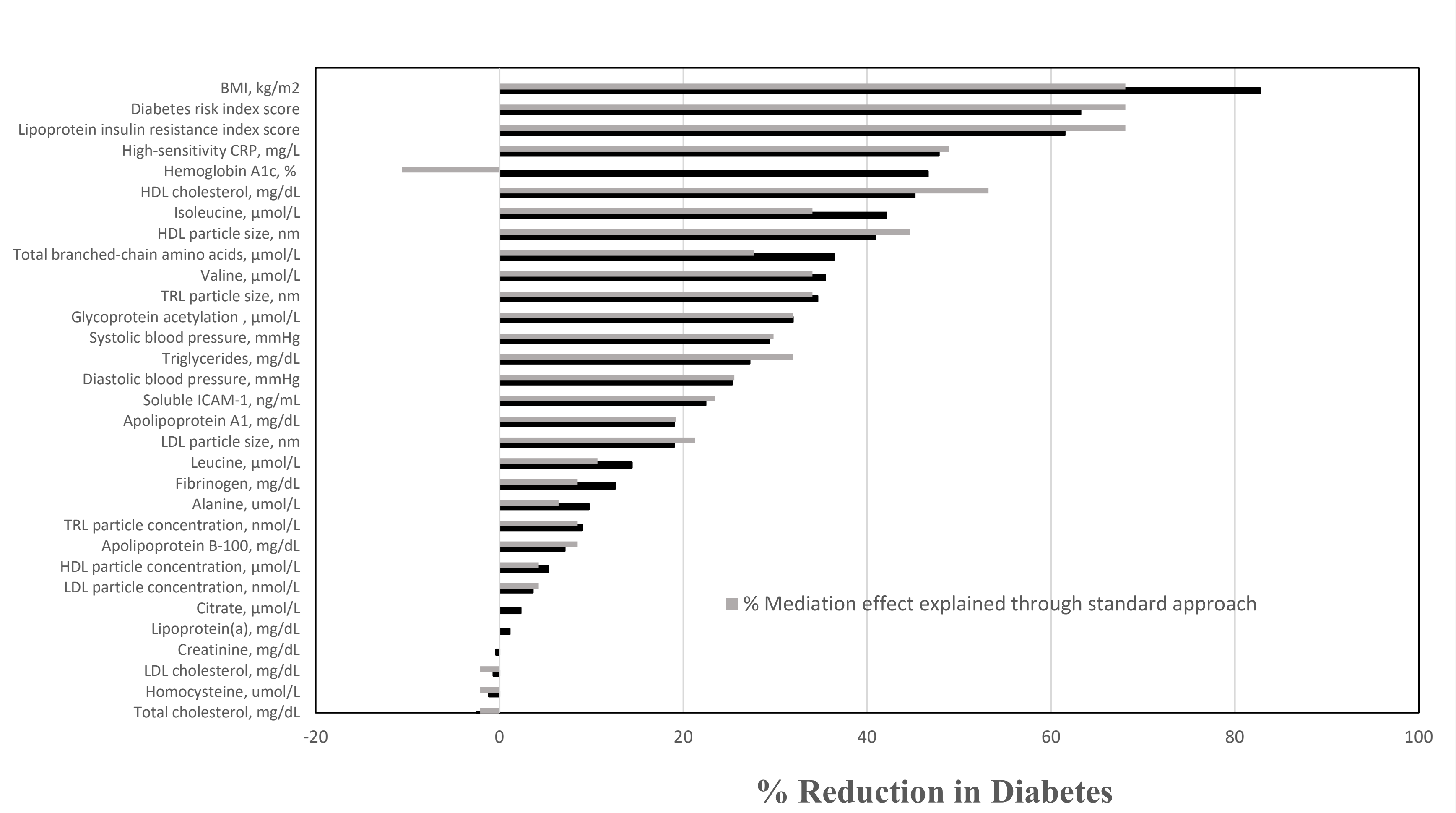

eFigure. Percentage Reduction in Incident Type 2 Diabetes Associated With Mediterranean Diet Explained by Potential Risk Mediators Using the Standard Mediation Approach and Counterfactual Framework Approach

The proportion of the risk reduction for MED ≥ 6 of Mediterranean diet intake (versus the reference group of MED 0-3).

© 2020 Ahmad S et al. *JAMA Network Open.*
